# 

**Published:** 1857-09

**Authors:** 


### CONTENTS.

ORIGINAL COMMUNICATIONS— PAGE.

Report on the Changes which take place in the Blood in the Continued Forms of Fever. By N. S. Davis, ........ ....... ........................ 391
Hints towards an inquiry, whether Tubercular tendency may be eradicated from the System, and by what means. By B. Woodward,...... 404
Cases in Practice, By E. K. Bowman,....................................... 409

PROCEEDINGS OF MEDICAL SOCIETIES—

Meeting of the Rock Island County Medical Society,........................ 413
Report of Representatives from Rock Island Medical Society on the proceedings of the State Medical Society. By E. K. Bowman,............ 414

SELECTIONS—

Dr. Edward Brown-Sequard's Experiments and Clinical Researches—Epilepsy—(continued),.................................................. 418
Prof. Gibson and Mr. Crawford the Sculptor........................... 424
Fiske Medical Prize Questions............................................ 426

BOOK NOTICES—

Manual of Physiology. By William Senhouse Kirkes.................... 427
Principles of Medicine. By Charles J. B. Williams........................ 427
Quinine in Fever. By Isaac Casselberry.................................. 428
The Use of Water in the Treatment of Fever. By Isaac Casselberry 428
Remarks on Fractures of the Scapula. By L. A. Dugas................. 428

EDITORIAL—

Medical Education.............................................................. 429
A New Pathy.................................................................... 432
DeWitt County Medical Society............................................. 432
Illinois State Medical Society Circular.................................. 433
Proceedings of the Medical Society at the Meeting in Ottawa in May last................................................................................ 435
Death of Dr. Marshall Hall................................................... 435

ADVERTISEMENTS—

To the Medical Profession, .................................................. 435
Medical College of Ohio, ..................................................... 437
Starling Medical College...................................................... 438

